# High-Quality Draft Genome Sequences of Marine Fish Gut *Bacillus* sp. Strains ABP1 and ABP2 with Nonstarch Polysaccharide Hydrolytic Potential

**DOI:** 10.1128/MRA.00077-20

**Published:** 2020-04-09

**Authors:** Cláudia R. Serra, Isabel M. Matas, Pedro Albuquerque, Antonio Muñoz-Mérida, Aires Oliva Teles, Paula Enes, Fernando Tavares

**Affiliations:** a CIIMAR–Centro Interdisciplinar de Investigação Marinha e Ambiental, Universidade do Porto, Matosinhos, Portugal; b CIBIO–Centro de Investigação em Biodiversidade e Recursos Genéticos, InBIO, Laboratório Associado, Universidade do Porto, Vairão, Portugal; c FCUP–Faculdade de Ciências, Departamento de Biologia, Universidade do Porto, Porto, Portugal; Georgia Institute of Technology

## Abstract

Here, we present the genome sequences of two environmental *Bacillus* strains with broad hydrolytic capacity toward different nonstarch polysaccharides (NSPs) that were isolated from the gut of marine fish fed NSP-rich diets. Several genes that may contribute to the NSP-degrading behavior were identified through *in silico* analysis.

## ANNOUNCEMENT

Spores of *Bacillus* species have been extensively explored as biocontrol agents, probiotics, and display systems (1–3). Although frequently found in soil, *Bacillus* spores have a ubiquitous distribution that includes the gastrointestinal tracts of different animals (4–6). Inside the animal gut, *Bacillus* strains can have a leading role in the degradation of complex food carbohydrates (7, 8).

Here, we present the genome sequences of two environmental *Bacillus* isolates (ABP1 and ABP2) that originated from the gut of European seabass (Dicentrarchus labrax), an important aquaculture marine fish species, fed on plant feedstuffs (PFs) (9). PFs are used as sustainable alternatives to fishmeal incorporation in aquafeeds (10), but their nutritive value is limited by nonstarch polysaccharides (NSPs), which are not metabolized by fish (11). Providing fish with gut bacteria capable of producing carbohydrate-active enzymes that hydrolyze NSPs has emerged as a potential strategy to overcome the limitations of PF diets. Among the isolates obtained from the gut of European seabass, strains ABP1 and ABP2 were particularly efficient as NSP hydrolyzers and showed probiotic potential in PF-enriched diets (9).

Genomic DNA was extracted from Luria-Bertani cultures (grown for 24 h at 37°C, 120 rpm) using the EZNA bacterial DNA purification kit (Omega Bio-Tek, GA, USA) and quantified with the Qubit 2.0 fluorometer (Invitrogen, OR, USA).

Shotgun genome sequencing was carried out at the Research and Testing Laboratory (Lubbock, TX, USA) using the PacBio RS II sequencer (Pacific Biosciences, CA, USA) after library preparation following the BluePippin size selection system. Totals of 78,219 and 96,855 reads (with mean read lengths of 13,383 and 15,478 bp) were obtained for ABP1 and ABP2, respectively. The raw sequences were assembled with Pacific Biosciences SMRT Analysis v2.3.0, using as a reference Bacillus subtilis subsp. *subtilis* strain 168 (GenBank accession number AL009126; 12). The total size of the assembly was 4,068 Mb (2 contigs, 4,063,450 bp and 4,608 bp) for ABP1 and 4,308 Mb (3 contigs, 25,608 bp, 2,201,652 bp, and 2,080,920 bp) for ABP2. A BLAST analysis against the RefSeq genome database (NCBI) in February 2016 (13) revealed that the best match for ABP1 was Bacillus subtilis subsp. *subtilis* strain BSP1 (accession number CP003695) (14), while for ABP2, the best match was *Bacillus* sp. LM 4-2 (accession number CP011101) (15).

Both assemblies were analyzed using the Rapid Annotation Subsystems Technology (RAST) server (16), and the final annotation was performed with Sma3s v2 (17). Default parameters were used for all software unless otherwise noted.

The 4,068,058-bp genome of strain ABP1 was found to contain 4,304 genes representing 4,184 protein-coding sequences, 10 complete sets of rRNAs (5S, 16S, and 23S rRNAs), and 86 tRNAs. Strain ABP2 contained a slightly bigger genome, with 4,308,180 bp and a total of 4,759 predicted genes encoding 4,643 proteins, 8 complete sets of rRNAs, and 82 tRNAs. The G+C contents of the genomes of ABP1 and ABP2 were estimated to be 43.9% and 43.4%, respectively.

Comparative to B. subtilis strain 168 (12), the genomes of ABP1 and ABP2 accommodate novel genes, some of which code for putative NSP-active hydrolases; genes involved in xylose and mannose metabolism were found in ABP1, while ABP2 contains a myo-inositol catabolic operon, which might contribute to catabolism of inositol phosphates in the marine environment or to their bioavailability (from PF diets) inside the fish gut. Taken together, the genomic sequences of strains ABP1 and ABP2 further corroborate their probiotic potential.

Both strains were deposited in the Spanish Colección Espanola de Cultivos Tipo (CECT), under the Budapest Treaty on the International Recognition of the Deposit of Microorganisms for the Purposes of Patent Procedure, and an international patent application was submitted (PTC/IB2019/059131).

### Data availability.

The whole-genome shotgun projects for strains ABP1 (CECT9675) and ABP2 (CECT9676) have been deposited at DDBJ/ENA/GenBank under accession numbers SIWZ00000000 and JAABUF000000000, respectively. The versions described in this paper are version numbers SIWZ00000000.1 and JAABUF000000000.1, respectively. The raw sequencing data are available at the Sequence Read Archive (SRA) under accession numbers SRR11091833 and SRR11091832, respectively.
